# The Impact of SARS-CoV-2 Infection in Patients with Inborn Errors of Immunity: the Experience of the Italian Primary Immunodeficiencies Network (IPINet)

**DOI:** 10.1007/s10875-022-01264-y

**Published:** 2022-04-20

**Authors:** Giuliana Giardino, Cinzia Milito, Vassilios Lougaris, Alessandra Punziano, Maria Carrabba, Francesco Cinetto, Riccardo Scarpa, Rosa Maria Dellepiane, Silvia Ricci, Beatrice Rivalta, Francesca Conti, Antonio Marzollo, Davide Firinu, Emilia Cirillo, Gianluca Lagnese, Caterina Cancrini, Baldassare Martire, Maria Giovanna Danieli, Andrea Pession, Angelo Vacca, Chiara Azzari, Giovanna Fabio, Annarosa Soresina, Carlo Agostini, Giuseppe Spadaro, Raffaele Badolato, Maria Pia Cicalese, Alessandro Aiuti, Alessandro Plebani, Isabella Quinti, Claudio Pignata

**Affiliations:** 1 Department of Translational Medical Sciences, Section of Pediatrics, Federico II University of Naples, Naples, Italy; 2 Department of Molecular Medicine, Sapienza University of Rome, Rome, Italy; 3 Department of Clinical and Experimental Sciences, Pediatrics Clinic and Institute for Molecular Medicine A. Nocivelli, University of Brescia, Brescia, Italy; 4 ASST-Spedali Civili Di Brescia, Brescia, Italy; 5 Department of Translational Medical Sciences, Center for Basic and Clinical Immunology Research, University of Naples Federico II, Naples, Italy; 6 Internal Medicine Department, Rare Disease Unit, Fondazione IRCCS Ca Granda Ospedale Maggiore Policlinico, Milan, Italy; 7 Rare Disease Referral Center, Internal Medicine 1, Ca Foncello Hospital, ULSS2 Marca Trevigiana, Treviso, Italy; 8 Department of Medicine-DIMED, University of Padova, Padua, Italy; 9 Department of Pediatrics, Fondazione IRCCS Ca Granda Ospedale Maggiore Policlinico, Milan, Italy; 10 Department of Health Sciences, University of Florence, Florence, Italy; 11 Department of Pediatrics, Immunology Unit, Meyer Children’s University Hospital, Florence, Italy; 12 Unit of Immunology and Infectious Diseases, Academic Department of Pediatrics, Bambino Gesù Children’s Hospital, Rome, Italy; 13 Department of Systems Medicine, University of Rome Tor Vergata, Rome, Italy; 14 Unit of Pediatrics, University of Bologna, St. Orsola University Hospital, Bologna, Italy; 15 Department of Women’s and Children’s Health, Pediatric Hematology-Oncology Unit, University of Padua, Padua, Italy; 16 Department of Medical Sciences and Public Health, University of Cagliari, Monserrato, Italy; 17 Unit of Pediatrics and Neonatology, “Monsignor A.R. Dimiccoli” Hospital, Barletta, Italy; 18 Clinica Medica, Dipartimento Di Scienze Cliniche E Molecolari, Università Politecnica Delle Marche E Azienda Ospedali Riuniti, Ancona, Italy; 19 Department of Biomedical Sciences and Human Oncology, Section of Internal Medicine and Clinical Oncology, University of Bari Medical School, Bari, Italy; 20 Pediatric Immunohematology and Bone Marrow Transplantation Unit, IRCCS San Raffaele Scientific Institute, Milan, Italy; 21 San Raffaele Telethon Institute for Gene Therapy (SR-Tiget), IRCCS San Raffaele Scientific Institute, Milan, Italy; 22 Vita-Salute San Raffaele University, Milan, Italy

**Keywords:** SARS-CoV-2, COVID-19, Inborn errors of immunity, Outcome, Seroconversion, Viral shedding

## Abstract

COVID-19 manifestations range from asymptomatic to life-threatening infections. The outcome in different inborn errors of immunity (IEI) is still a matter of debate. In this retrospective study, we describe the experience of the of the Italian Primary Immunodeficiencies Network (IPINet). Sixteen reference centers for adult or pediatric IEI were involved. One hundred fourteen patients were enrolled including 35 pediatric and 79 adult patients. Median age was 32 years, and male-to-female ratio was 1.5:1. The most common IEI were 22q11.2 deletion syndrome in children (26%) and common variable immunodeficiency (CVID) in adults (65%). Ninety-one patients did not require hospital admission, and among these, 33 were asymptomatic. Hospitalization rate was 20.17%. Older age (*p* 0.004) and chronic lung disease (*p* 0.0008) represented risk factors for hospitalization. Hospitalized patients mainly included adults suffering from humoral immunodeficiencies requiring immunoglobulin replacement therapy and as expected had lower B cell counts compared to non-hospitalized patients. Infection fatality rate in the whole cohort was 3.5%. Seroconversion was observed is 86.6% of the patients evaluated and in 83.3% of CVID patients. 16.85% of the patients reported long-lasting COVID symptoms. All but one patient with prolonged symptoms were under IgRT. The fatality rate observed in IEI was slightly similar to the general population. The age of the patients who did not survive was lower compared to the general population, and the age stratified mortality in the 50–60 age range considerable exceeded the mortality from 50 to 60 age group of the Italian population (14.3 vs 0.6%; *p* < 0.0001). We hypothesize that this is due to the fact that comorbidities in IEI patients are very common and usually appear early in life.

**Supplementary Information:**

The online version contains supplementary material available at 10.1007/s10875-022-01264-y.

## Introduction

During the last 2 years, severe acute respiratory syndrome coronavirus 2 (SARS-CoV-2) has spread worldwide, causing about 5 million deaths. Clinical manifestations are very variable, going from a completely asymptomatic infection to multiorgan failure and death [1]. The main risk factors of worse outcome in the general population include older age, male sex, and pre-existing comorbidities. Recent studies suggest that the clinical course of the infection may not be different in patient with inborn errors of immunity (IEI) compared to the general population [2–4]. However, since IEI are a heterogeneous group of disorders including more than 400 different clinical conditions [5], more studies are necessary to better define the outcome of the infection in the different forms. Specific immune defects may serve as an *experimentum naturae* suggesting the role of specific branches of the immunity in predisposing or even protecting from a worse outcome and a better clarification of the role of different branches of the immunity in the response to SARS-CoV-2 may pave the way for the development of novel targeted treatments. Many aspects of the infection still need to be clarified in this syndrome. These include the ability to clear the infection in different IEI and the ability to develop an effective immunological memory post-infection.

In this paper, we describe the clinical course and outcome in a cohort of IEI patients followed at centers of the of the Italian Primary Immunodeficiencies Network (IPINet) who experienced SARS-CoV-2 infection between March 2020 and April 2021. The aim of the study is to better define the clinical course of the infection and define risk factors for a worse outcome in a large cohort of patients.

## Material and Methods

Patients in follow-up at IPINet centers who experienced SARS-CoV-2 infection between March 2020 and April 2021 were included in the study. SARS-CoV-2 infection was confirmed by RT-PCR on the nasopharyngeal swab or through antibody testing. For each patient, data were collected retrospectively from the clinical records. The survey was sent to 16 Italian reference centers for adult (eight centers: Milano, Brescia, Padova, Ancona, Roma, Napoli, Bari, Cagliari) or pediatric (eight centers: Milano, Brescia, Padova, Bologna, Firenze, Roma, Napoli, Bari) IEI. Most of the cases have been already included in another study on the IPINet cohort focusing on incidence, outcome, and infection-fatality rate [6]. A total of 33 patients included in the previous study were not included in the current study since the referring center decided not to take part in the second study or because detailed information on the clinical course of the infection was missing. Moreover, compared to the previous study, the current study includes 16 novel patients identified after the conclusion of the previous study. These include patients *n*° 13, 18, 25, 38, 40–45, 58, 64, 86, and 99–101 in the supplemental table. In this study, we performed an in-depth evaluation including detailed information on the type of underlying IEI, sex, age, comorbidities, and ongoing therapies along with baseline immunological and lung function assessment. As for the baseline immunological assessment, information collected included immunoglobulin levels, number and percentage of white blood cells (WBC), neutrophils, lymphocytes, T lymphocytes (CD3 +), helper T cells (CD4 +), cytotoxic T cells (CD8 +), B cells (CD19 +), and NK cells (CD16 + CD56 +). As for the lung function, information collected included baseline SatO2% and pre-existing structural lung damage (atelectasis, bronchiectasis, interstitial lung disease, or nodules). To evaluate the duration of the viral shedding, the dates of the first positive and first negative nasopharyngeal swab were recorded. The patients were then stratified according to hospitalization. For patients not requiring hospitalization, information collected included body temperature, resting SatO2%, SatO2% after 6-min walking test (6MWT), and type and duration of the symptoms including cough, headache, sore throat, nasal obstruction, anosmia/ageusia, myalgia, nausea/vomiting, diarrhea, loss of appetite, conjunctivitis, dyspnea, increased expectorate, and hemoptoe. The average time to recovery from COVID-19 is highly variable and depends on age and pre-existing comorbidities and illness severity [7, 8]. In individuals with mild infection, recovery time is usually about 2 weeks, whereas in individuals with severe disease, longer time was observed [7, 8]. We evaluated the percentage and the clinical feature of patients with COVID symptoms lasting more than 3 weeks. In the group of patients requiring hospitalization, when available, we collected data about PCR, LDH, ferritin, IL-6, D-dimer, procalcitonin, platelet count, number and percentage of white blood cells (WBC), neutrophils, lymphocytes, T lymphocytes (CD3 +), helper T cells (CD4 +), cytotoxic T cells (CD8 +), B cells (CD19 +), NK cells (CD16 + CD56 +), SatO2%, degree of respiratory involvement, presence of pulmonary embolism or deep vein thrombosis, need of ICU admission, oxygen requirement, requirement of mechanical ventilation or extracorporeal membrane oxygenation (ECMO), complications, and duration of the hospitalization and outcome. Finally, according to IEI type, information on the seroconversion was collected.

### Statistical Analysis

The statistical software packages GraphPad and Excel were used for the statistical analyses and to produce all the graphs. All the values are showed as means ± standard deviation. Two tail *t* test for independent samples and Fisher’s exact or chi-square test were used to compare means and proportions, respectively. Values of *P* < 0.05 were considered statistically significant.

## Results

### Baseline Clinical and Immunological Features of the Study Population

**Fig. 1:**
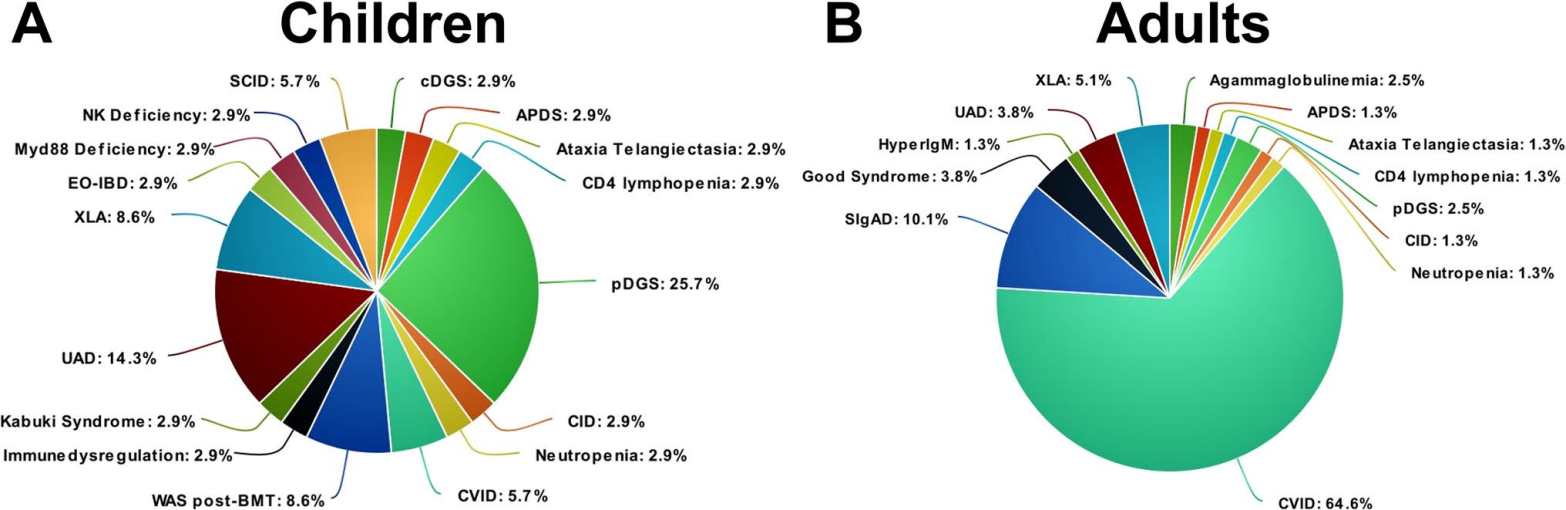
Distribution of different IEI in pediatric and adult cohorts. **a** 22q11.2DS represented the most common IEI (26%) in the pediatric cohort, followed by unclassified antibody deficiency (UAD, 14%), X-linked agammaglobulinemia (XLA, 9%), and Wiskott-Aldrich (WAS, 9%). **b** In the adult cohort, the most common IEI was CVID (65%) followed by selective IgA deficiency (SIgAD, 10%) and XLA (5%)

**Fig. 2:**
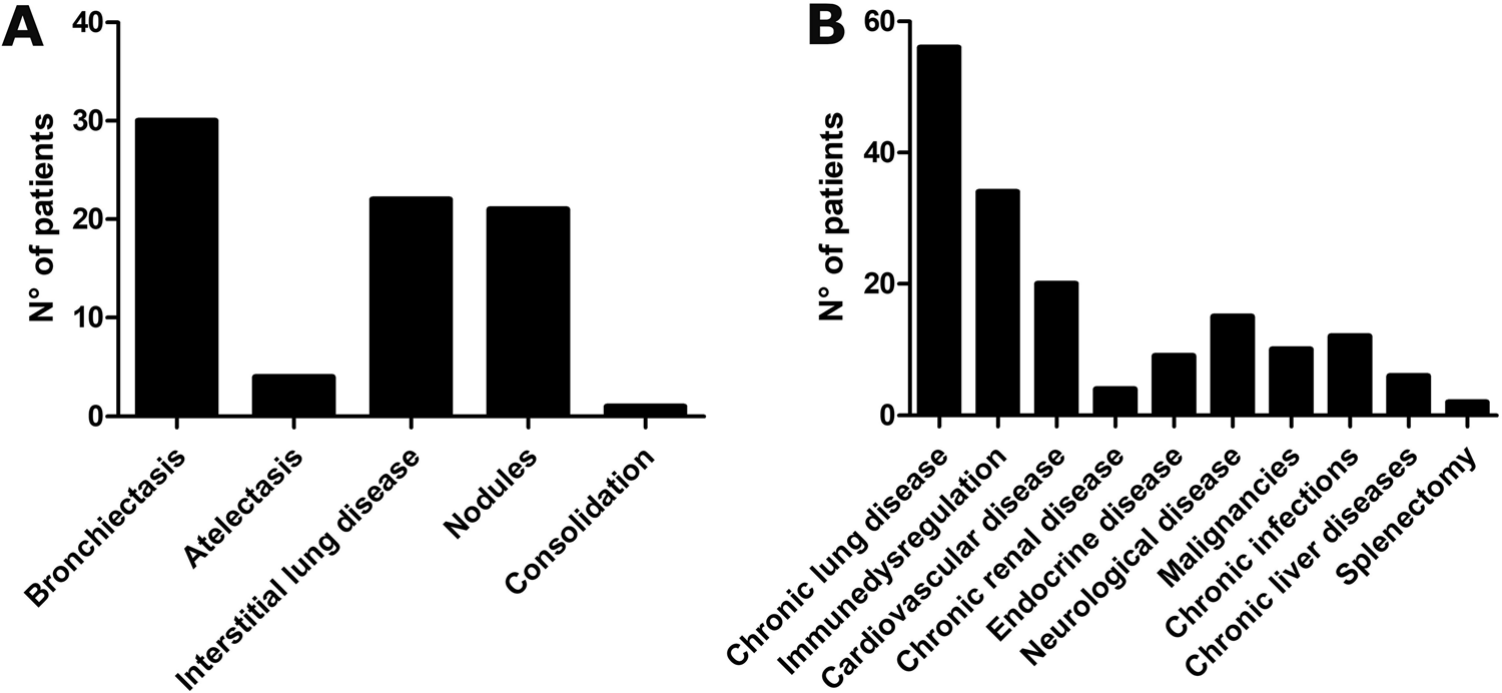
Baseline structural lung alterations and comorbidities. **a** The most common baseline structural lung alterations included bronchiectasis, interstitial lung disease, and nodules. **b** The most common comorbidities included chronic lung disease, immune dysregulation, and heart disease

One hundred fourteen patients were included into the study. In most of the cases (108/114), the diagnosis of SARS-CoV-2 infection was confirmed through RT-PCR on the nasopharyngeal swab. In the remaining cases (6/114), a previous infection was identified during routine antibody testing during follow-up visits. Median age was 32 years (mean 33.2 ± 20.1 years), and male-to-female ratio was 1.5:1. The cohort included 35 pediatric and 79 adult patients. In the pediatric cohort, 22q11.2 deletion syndrome (DS) represented the most common IEI (26%), followed by unclassified antibody deficiency (UAD, 14%), X-linked agammaglobulinemia (XLA, 9%), and Wiskott-Aldrich (WAS, 9%) (Fig. 1a). In the adult cohort, the most common IEI was common variable immunodeficiency (CVID, 65%) followed by selective IgA deficiency (SIgAD, 10%) and XLA (5%) (Fig. 1b). Three patients had received bone marrow transplantation (BMT), 2 gene therapy, and one thymus transplant. Thirty-one patients were receiving intravenous immunoglobulin (IVIg) replacement therapy, 42 subcutaneous immunoglobulin (SCIg), 16 antibiotic prophylaxis, 12 immunosuppressive treatment including prednisone (5 patients), mycophenolate mofetil (2 patients), mesalazine (2 patients), and others (Supplemental table). Baseline immunological evaluation revealed neutropenia in 4/111 patients, lymphopenia in 20/112 patients, involving the CD3 + compartment in 22/92 patients, the CD4 + compartment in 15/92, the CD8 + compartment in 11/92, and the CD19 + compartment in 39/92. Seven out of 79 patients had impaired baseline SatO2% ranging from 85 to 96%. Fifty-four out of 68 studied patients had baseline structural lung alterations, including bronchiectasis in 30 cases, atelectasis in 4, interstitial lung disease in 22, nodules in 21, and consolidation in 1 (Fig. 2a). Eighteen patients had more than 1 lung alteration. Two patients in which a baseline lung CT scan was not available had a history of chronic obstructive lung disease. The most common comorbidities included chronic lung disease (56 cases), immune dysregulation (34 cases), heart disease (20 cases), neurological disease (14 cases), and chronic infection (12 cases). The remaining comorbidities are summarized in Fig. 2b.

### Symptoms in Patients Who Did Not Require Hospital Admission

**Fig. 3:**
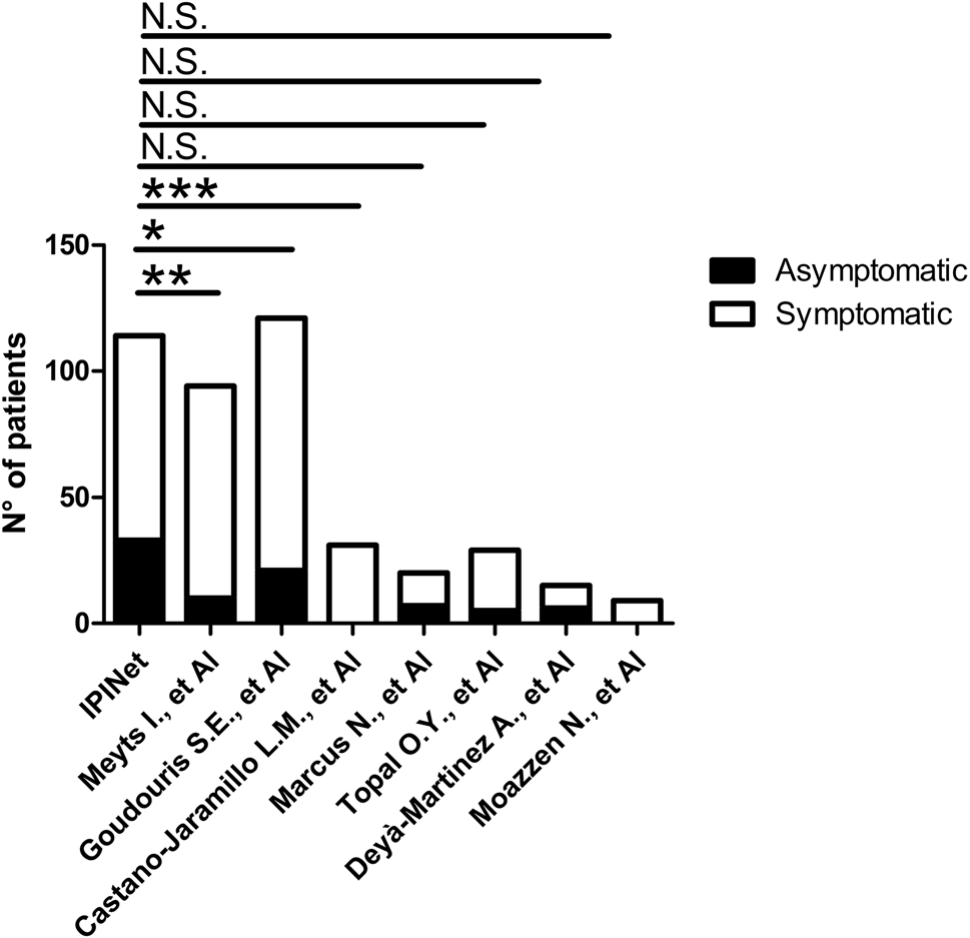
Comparison of the percentage of asymptomatic patients among our cohort and previous studies from the literature. *p ≤ 0.05; **p ≤ 0.01; ***p ≤ 0.001

**Fig. 4:**
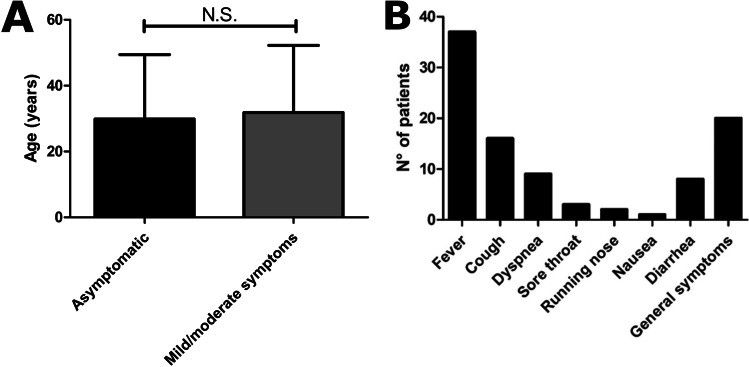
Clinical course in non-hospitalized patients. **a** Mean age in asymptomatic patients vs patients with mild-moderate symptoms. **b** Main clinical symptoms in non-hospitalized patients

Ninety-one patients did not require hospital admission. Information on the clinical course of the infection was available for 80/91 patients. Of these, 33 patients were asymptomatic. These included patients affected with different IEI including CVID (14), UAD (3), agammaglobulinemia (3), CID (2), 22q11.2DS (2), WAS post-BMT (2), APDS (1), ataxia telangiectasia (1), hyperIgM (1), immune dysregulation (1), Myd88 deficiency (1), post-HSCT SCID (1), and SIgAD (1). Mean age in asymptomatic patients was 29.85 ± 19.56 years (median 24 years; range 1.4–65 years), and male-to-female ratio was 3.6:1. We compared the rate of asymptomatic patients in our cohort with that observed in cohorts of patients with IEI previously published. Interestingly, the rate of asymptomatic patients in our cohort was significantly higher compared to the international cohort published by Meyts et al. [2], the Brazilian [9], and Mexican cohorts [10], while no difference was observed with Israelian, Turkish, Spanish, and Iranian cohorts (Fig. 3). Within the non-hospitalized group, 47 patients had mild symptoms. Mean age in this group was 33.28 ± 21.10 years (median 31 years), and male-to-female ratio was 1:1.04. The mean age was not significantly different between asymptomatic and patients with mild/moderate symptoms (Fig. 4a). The most common symptom was fever observed in 37 patients (Fig. 4b). In 25 cases, temperatures were recorded at home and maximum temperatures ranged from 37 to 39.5 °C (mean 38 ± 0.64 °C; median 38 °C). The mean duration of the fever was 12.54 ± 16.68 days (median 7 days). Eighteen patients reported respiratory symptoms as cough in 16 patients, dyspnea in 9 patients, sore throat in 3 patients, and running nose in 2 patients (Fig. 4b). Resting SatO2 was measured at home in 33 patients and ranged from 89 to 100% (mean 96.82 ± 3.20%; median 97.5%). SatO2 after 6MWT was available for 8 patients and ranged from 93 to 98% (mean 97.3 ± 1.63%; median 98%). The mean duration of cough was 10.86 ± 9.22 days (median 7 days). Nine patients reported gastrointestinal symptoms (GI) including nausea in 1 patient and diarrhea in 8 patients (Fig. 4b). The mean duration of GI symptoms was 6.16 ± 4.95 days (median 4.5 days). General symptoms are reported in 20 patients, including asthenia, myalgia, anosmia, and ageusia (Fig. 4b). Five non-hospitalized patients experienced a prolonged infection, defined as the persistence of symptoms for more than 3 weeks [7, 8, 11, 12]. Mean duration of prolonged infection in non-hospitalized patients was 41 ± 27.47 days (range 25–90 days). In 2 cases, the only symptom lasting more than 3 weeks was fever, while in 3 cases, fever was associated with respiratory and general symptoms. In one of these cases, also diarrhea was reported.

### Clinical and Laboratory Features of Patients Requiring Hospital Admission

**Fig. 5:**
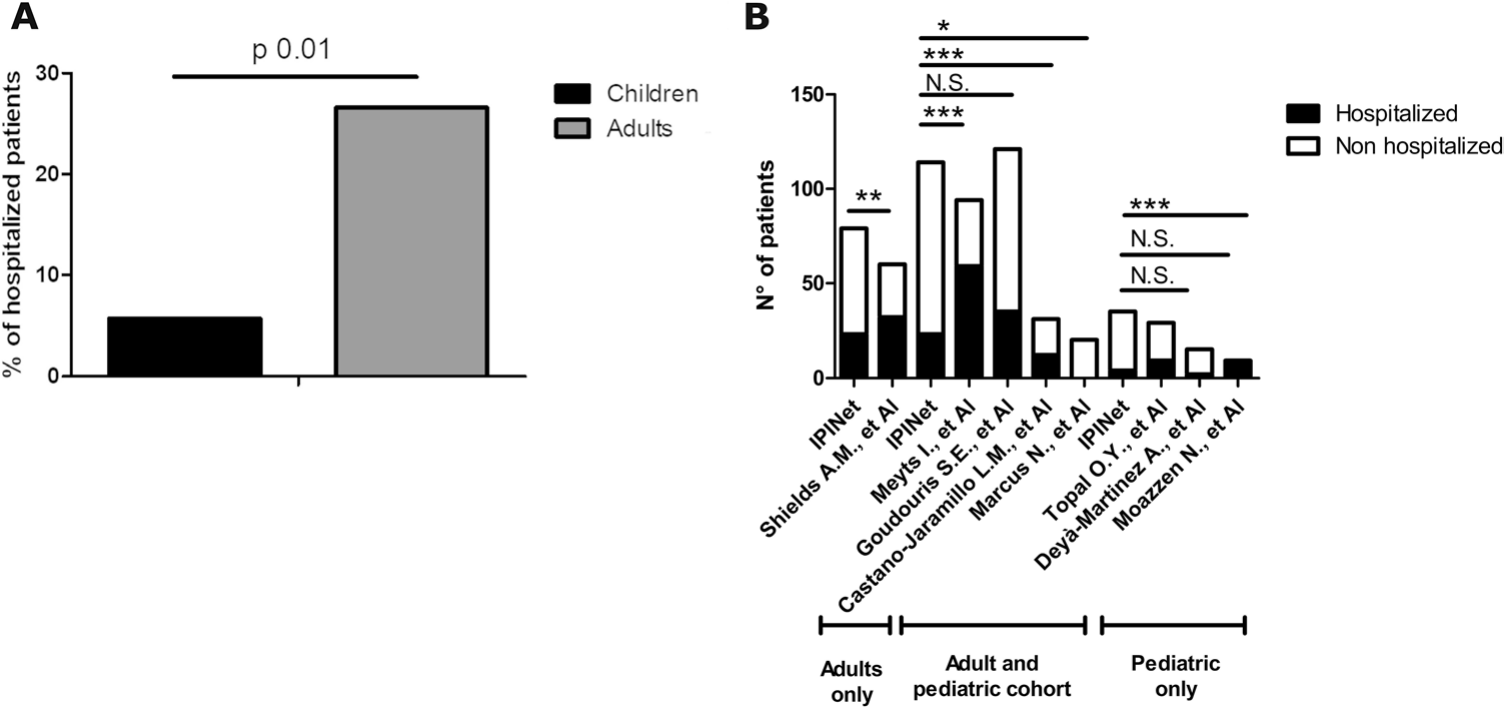
Hospitalization rate. **a** Hospitalization rate in pediatric vs adult cohort. **b** Comparison of the hospitalization rate among our cohort and previous studies from the literature

**Fig. 6:**
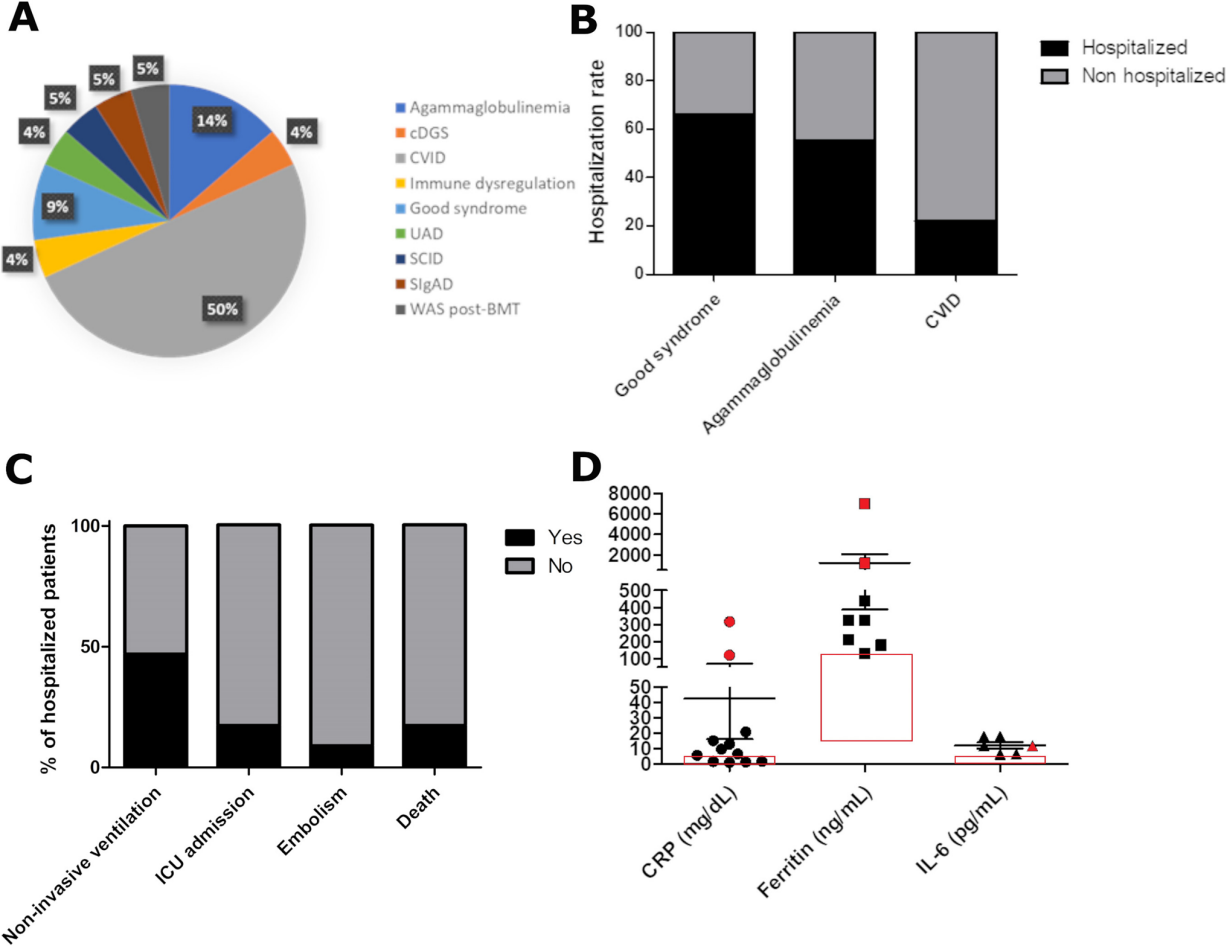
Clinical features of the hospitalized patients. **a** Types of IEI in the hospitalized patients. **b** Hospitalization rate in the 3 most represented IEI. **c** Complications and outcome in hospitalized patients. **d** C reactive protein (CRP), ferritin, and IL-6 levels in hospitalized patients. Red symbols indicate ICU patients. Red boxes indicate reference intervals

Twenty-five patients required hospital admission (21.92%). These included 2 children who were admitted for medical reasons different from COVID-19, who were excluded from the following analysis. Thus, the hospitalization rate was 20.17%. As expected, the hospitalization rate was higher in the adult compared to the pediatric cohort (26.58 vs 5.7%, *p* 0.01) (Fig. 5a). We compared the hospitalization rate in our cohort with that observed in IEI cohorts of patients previously published. When we compared it with the UK cohort, we only included adult cases since the UK cohort mostly included adults vice versa when we compared it with the Turkey, Spain, and Iran cohorts, we only included pediatric cases. Interestingly, the hospitalization rate in our cohort was significantly lower compared to the international cohort published by Meyts et al. [2], the UK [13], the Mexican [10], and the Iranian cohorts, while we did not find any difference with the Brazilian [9], the Turkish, and the Spanish cohorts (Fig. 5b). In Israelian cohort, the hospitalization rate was significantly lower compared to our cohort [14]. Hospitalized patients mainly included patients with humoral immunodeficiencies and, in particular, CVID (50% of the cases) (Fig. 6a). The hospitalization rate varied among different IEIs. In particular, the highest hospitalization rate was observed in Good’s syndrome (66%), agammaglobulinemia (55%), and CVID (22%) (Fig. 6b). The mean duration of hospital admission was 23.23 ± 16.73 days. Mean age in hospitalized patients was 40.39 ± 19.43 years (ranging from 1 to 80 years; median 47 years), and male-to-female ratio was 2.83:1. Detailed information on the course of the disease was available for 19 patients. Ten out of 19 patients suffered from prolonged infection, characterized only by fever in one case, only respiratory symptoms in another case, and fever and respiratory and general symptoms in 8 cases. In one case, diarrhea was reported. The mean duration of the symptoms in these cases was 45.4 ± 12.25 days (range 30–60 days). The prevalence of prolonged infection in the whole cohort was 16.85%, and the occurrence of prolonged infection was significantly higher in hospitalized vs non-hospitalized patients (52.63 vs 5.5%, *p* < 0.0001). These included patients with different IEI, namely CVID (7), XLA (2), SCID (NBAS deficiency) (1), cDGS (1), Good’s syndrome (1), UAD (2), and SIgAD (1). All the patients with prolonged infection, except the patient with SIgAD deficiency, were under IgRT. The prevalence of prolonged infection was 16% in CVID and 40% in XLA.

Nine out of 19 hospitalized patients developed respiratory failure requiring oxygen administration/non-invasive ventilation (Fig. 6c). Four patients required ICU admission for invasive ventilation, and none of them required ECMO (Fig. 6c). Two patients developed pulmonary embolism and/or deep vein embolism (Fig. 6c). CRP, ferritin, and IL-6 values were available for 12, 8, and 5 patients, respectively, and ranged from 0.9 to 316.8 mg/L, 129 to 7020 ng/mL, and from 6.46 to 18 pg/mL, respectively (median 8.04 mg/L, 326 ng/mL, and 11.8 pg/mL, respectively) (Fig. 6d). Three out of 10 patients had lymphopenia and 3 had thrombocytopenia. Mean age of patients admitted to ICU was 53 ± 7.61 years, and male-to-female ratio was 3:1. All the 4 patients requiring ICU admission suffered from CVID. Two out of 4 patients requiring ICU had a history of chronic lung disease including atelectasis in 1 case and interstitial lung disease with nodules in the other one. Other comorbidities included eosinophilic granulomatosis with polyangiitis, hypertension, and impaired glucose tolerance in one patient; chronic immune-mediated thrombocytopenia in the second; and chronic sinusitis in the third. One of the patients requiring ICU admission had no comorbidities. Two out of 4 hospitalized patients admitted to ICU died. A total of 4 patients died during hospital admission. The inpatient fatality rate was 17.39% (Fig. 6c), and the fatality rate in the whole cohort was 3.5%. Male-to-female ratio in patients who died was 2.5:1, and mean age was 48.00 ± 6.26. IEI in patients who died included CVID (3 patients) and Good’s syndrome (1 patients). Fatality rate was 5.7% for CVID and 33.3% for Good’s syndrome. As reported in the general population, CRP, ferritin, and IL-6 levels were very high in patients requiring ICU admission, being 120 and 316.8 mg/L, 7020 and 1167 ng/mL, and 11.8 pg/mL, respectively. Complications in hospitalized patients included hypocalcemia and tonic–clonic seizures in a child with cDGS, worsening of pulmonary hypertension in one case and mental confusion in another.

### General Outcome, Immunological, and Non-immunological Risk Factors for a Worse Outcome

As expected, older age represented a risk factor for hospitalization. In fact, hospitalization rate was higher in adults compared to pediatric patients (26.58 vs 5.7%, *p* 0.01; OR 5.9741, CI 1.3170 to 27.0996), and non-hospitalized patients were significantly younger than hospitalized patients (30.85 ± 20.08 vs 43.37 ± 17.16 years, *p* 0.004). Moreover, patients who died were older than survivors (48.00 ± 6.26 vs 34.97 ± 2.19 years, *p* 0.204) even though this difference was not statistically significant. Immunological risk factors for hospital admission were represented by lower baseline B cell count and IgRT. However, this is likely due to the fact that hospitalized patients were mainly older and suffered from humoral immunodeficiencies. On the contrary, baseline lymphocyte, neutrophil, CD3 + lymphocyte, CD4 + lymphocyte, CD8 + lymphocyte, and CD16 + CD56 + counts were comparable between hospitalized and non-hospitalized patients and between survivors and non-survivors (Table 1). Male-to-female ratio was higher in hospitalized patients, even though this difference was not statistically significant.

**Table 1:** Risk factors for hospitalization and death

Variable	Not hospitalized	Hospitalized	OR for hospitalization (95% CI)	*p* value	Survived	Died	OR for mortality (95% CI)	*p* value
*n*	91	23	-	-	-	-	-	-
Age	30.85 ± 20.08	43.37 ± 17.16	-	0.004	34.97 ± 2.19	48.00 ± 6.26	-	0.204
Baseline lymphocyte count (adults)	3439.19 ± 1097.61	4007.14 ± 1911.23	-	0.2102	1772.52 1015.02	1677.5 ± 831.4	-	0.838
Baseline neutrophil count (adults)	1641.01 ± 781.73	2117.61 ± 1414.44	-	0.1555	3488.97 ± 1134.28	5500 ± 3473.87	-	0.331
Baseline CD3 + count	1265.60 ± 748.11	1443.12 ± 893.88	-	0.45	1299.37 ± 773.19	1269.7 ± 1006.46	-	0.964
Baseline CD4 + count	678.33 ± 444,14	719.76 ± 527.56	-	0.76	694.1 ± 49.32	450.0 ± 114.3	-	0.366
Baseline CD8 + count	470.25 ± 308.02	625.86 ± 574.88	-	0.32	485.68 ± 349.71	1004.5 ± 972.27	-	-
Baseline CD19 + count	219.26 ± 314.63	61.60 ± 141.88	-	0.004	219.6 ± 34.98	228.0 ± 228.0	-	0.970
Baseline CD16 + CD56 + count	211.42 ± 203.29	259.93 ± 649.81	-	0.78	222.76 ± 321.94	91.5 ± 37.47	-	-
Sex (M:F)	1.28:1	2.83:1	2.210 (0.7963–6.134)	0.15	-	-	-	-
IgRT	56/91	20/23	4.1667 (1.1528–15.0596)	0.0253	72/110	4/4	0.2092 (0.01097–3.992)	0.2994
Prophylactic antibiotics	12/91	5/23	1.8287 (0.5721–5.8458)	0.3305	16/110	1/4	0.5106 (0.04993–5.222)	0.4808
Current immunosuppression	7/91	4/23	2.5263 (0.6711–9.5098)	0.1706	10/110	1/4	0.3000 (0.02846–3.162)	0.3374
Chronic lung disease	38/91	19/23	6.6250 (2.0853–21.0474)	0.0008	54/110	3/4	0.3214 (0.03241 to 3.188)	0.6183
Cardiovascular disease	16/91	5/23	1.3021 (0.4213–4.0240)	0.6466	20/110	1/4	0.6667 (0.06584–6.750)	0.5624
Chronic liver disease	3/91	1/23	1.3333 (0.1322–13.4451)	0.8072	4/110	0/4	0.3803 (0.01764–8.200)	1.000

*OR* odds ratio; *CI* confidence interval; *IgRT* immunoglobulin replacement therapy

## Seroconversion and Viral Shedding

Serology was evaluated in 30 patients, and seroconversion was documented in 26 patients (2 patients with WAS post-GT, 2 patients with UAD not requiring IgRT, 5 patients with CVID under IgRT, 1 patient with immune dysregulation, 1 patient with Myd88 deficiency, 1 patient with hyperIgM syndrome, 4 patients with 22q11.2DS, 1 patient with ataxia telangiectasia, 1 patient with SCID post-HSCT, 1 patient with SCID (NBAS deficiency), 1 patient with neutropenia, 2 patients with combined immunodeficiency, 1 patient with Good’s syndrome under IgRT, 1 patient with NK deficiency, 1 patient with EO-IBD, and 1 patient with SIgAD). In 4 patients, there was no seroconversion, and these included patients with Good’s syndrome, cDGS, agammaglobulinemia, and CVID. All the patients without seroconversion, except for the patient with Good’s syndrome, were under IgRT. Seroconversion was observed in 5/6 patients with CVID (83.3%).

**Fig. 7:**
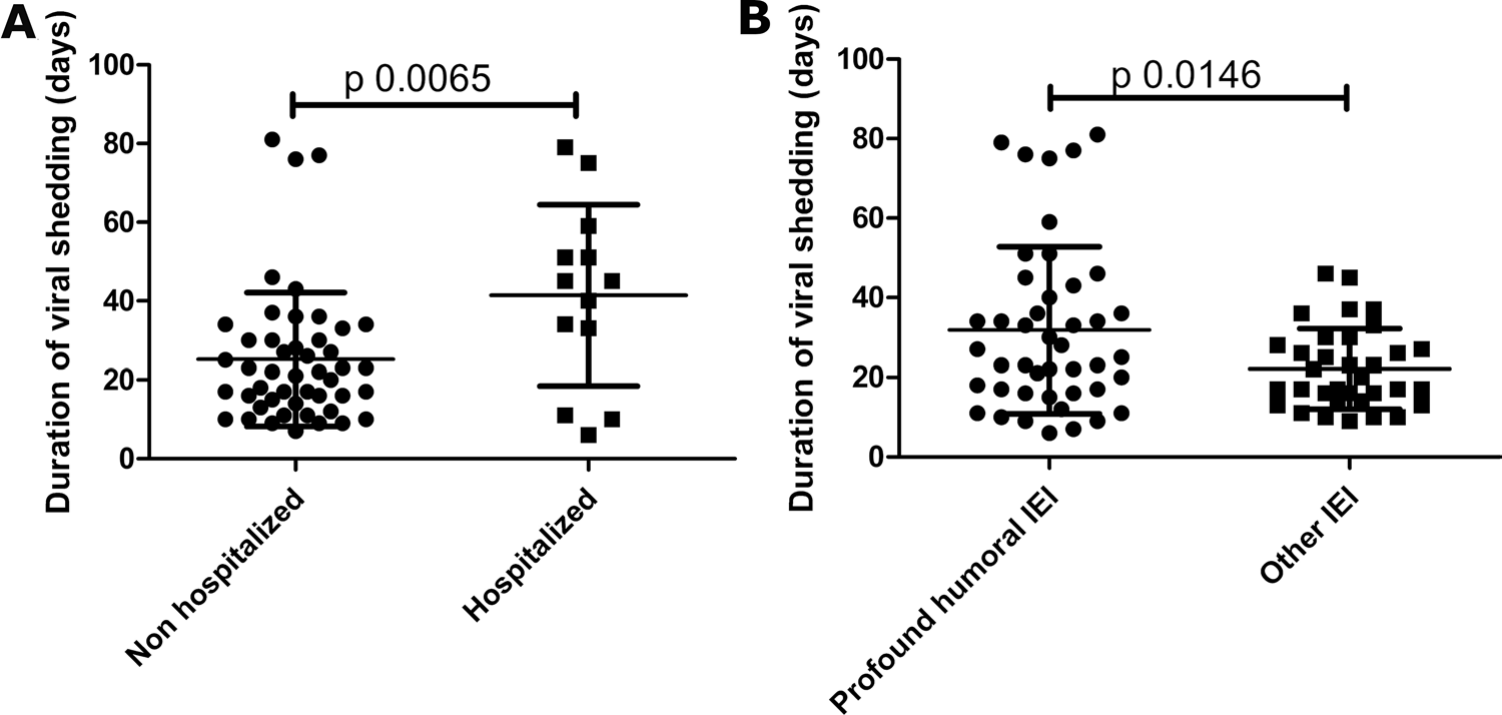
Risk factors for longer viral shedding. **a** Comparison of the viral shedding duration among non-hospitalized and hospitalized patients. **b** Comparison of the viral shedding duration among patients with profound humoral IEI vs other IEI

Information on the duration of viral shedding was available for 60 patients. The mean duration of viral shedding was 28.71 ± 19.42 days (median 23 days; range 6–81 days). Viral shedding was longer in hospitalized compared to non-hospitalized patients (41.46 ± 23.00 vs 25.19 ± 16.95, *p* 0.0065) (Fig. 7a) and in patients with profound humoral IEI compared to patients with other IEI (31.84 ± 20.93 vs 22.09 ± 10.08 days, *p* 0.0146) (Fig. 7b).

### Treatment of SARS-CoV-2 Infection

The treatment of SARS-CoV-2 infection varied according to the period of observation. Detailed information on the treatment was available for 13/19 hospitalized patients. Six patients were treated with antivirals including remdesivir in 4 cases and lopinavir/ritonavir in 3 cases. Three patients were treated with hydroxychloroquine. Nine patients were treated with antibiotics including in most cases azithromycin (5 cases). Low molecular weight heparin was used in 7 cases. Immunosuppressive drugs were used in 10 cases and included steroids in 10 cases, anakinra in one pediatric patient prolonged infection and tocilizumab in one case. Hyperimmune plasma was used to treat 3 patients, two of whom affected with prolonged infection and casirivimab/imdevimab was used in 2 patients with relapsing COVID.

## Discussion

In the period March 2020 to April 2021, a total of 114 patients infected by SARS-CoV-2, including both children and adults with IEI, were identified in the enrolled centers. Most of the pediatric patients suffered from 22q11.2DS, while most of the adults suffered from humoral immunodeficiencies and in particular CVID. This distribution parallels the distribution of IEI cases in the two groups.

Differently from some of the previously published IEI cohorts, most of the patients (80%) in the present study were managed at home and did not require hospital admission [2, 13, 15]. The hospitalization rate observed in our study was significantly lower compared to some of the previous studies [2, 9, 10, 13]. No difference in the inpatient mortality rate and the ICU admission rate was observed in our cohort compared to previous studies with higher hospitalization rate, implying that the criteria for the hospital admission were similar in the different cohorts. This variability could reflect the knowledge gained during the pandemic and different treatment modalities that are being used currently versus earlier in the pandemic. In our cohort, almost 30% were completely asymptomatic and 50% developed mild to moderate symptoms, and the rate of asymptomatic patients was higher compared to some of the previous studies [2, 9, 10, 13], while no difference was observed with Israelian [14], Turkish [16], Spanish [17], and Iranian cohorts [18]. The variability of the prevalence of asymptomatic cases possibly reflects the different access to testing in different phases of the pandemics and across different countries. The higher rate of asymptomatic patients in our cohort is, indeed, likely due to the greater screening of individuals without symptoms [19]. In fact, after COVID-19 outbreak in Lombardy region, SARS-CoV-2 screening became mandatory to access most Italian hospitals as inpatients or outpatients, to avoid hospital outbreaks. SARS-CoV-2 screening was especially required when aerosol generating procedures were performed. Since most IEI patients require very frequent hospital admissions, it is more likely for asymptomatic IEI patients to be identified. An increasing rate of asymptomatic patients during different phases of the pandemics was also observed in the general population, since at the beginning of the pandemics, the access to the testing was mainly addressed to the most symptomatic patients.

In our cohort, asymptomatic patients included patients with different IEI. Interestingly, among the asymptomatic patients, we also identified a patient with Myd88 deficiency. The patient is of Romani ethnicity and is affected with a classical early onset form caused by a homozygous c.192_194delGGA (p.Glu66del) mutation, associated with a clinical history of severe infections. This was quite surprising in that innate immune response is critical for recognizing and controlling infections through the release of cytokines and chemochines. In SARS-CoV-2 infection, hyperactive cytokine release and cytokine storm are associated with the development of severe forms. A recent study shows that TLR2 and Myd88 are implicated in SARS-CoV-2-induced inflammatory response and are associated with COVID-19 disease severity [20]. This would suggest that Myd88 deficiency may dampen the massive cytokine release associated with the severe forms. Nonetheless, in a recent study on a pediatric cohort of IEI patients, two young brothers with Myd88 deficiency developed pneumonia requiring hospital admission in both cases and oxygen administration in one case [17].

Mean age of the asymptomatic patients was not significantly lower compared to that of patients with mild-moderate symptoms. The most common symptom reported in patients not requiring hospital admission was fever followed by general symptoms and respiratory symptoms. Gastrointestinal symptoms were more rarely observed, and, differently from what observed in the general population, there was no difference among adults and children in the incidence of gastrointestinal symptoms [21, 22]. In our pediatric cohort, we did not identify any case of multisystem inflammatory syndrome in children (MIS-C).

As expected, hospitalization rate was higher in adults than in children, and in the whole cohort, mean age of hospitalized patients was significantly higher than non-hospitalized. In the pediatric cohort, only 2 children required hospital admission: a 1-year-old patient with cDGS and a 2.75-year-old child with NBAS deficiency [23]. Both patients developed COVID-19 symptoms lasting more than 4 weeks, and both recovered without sequelae. Most of the adult patients suffered from humoral immunodeficiencies, and in particular, half of them had CVID. The highest hospitalization rate was observed for Good’s syndrome followed by agammaglobulinemia and CVID. The clinical course of the infection in hospitalized patients was comparable between our study and the international study reported by Meyts et al. [2]. In particular, among the patients requiring hospital admission, the percentage of patients requiring non-invasive ventilation or ICU admission and the inpatient mortality rate were comparable between the two studies. When we compared the baseline laboratory parameters between hospitalized and non-hospitalized patients, we found that B cell count was significantly lower in the former group. However, it should be noted that hospitalized were older than non-hospitalized patients and that 82% of the hospitalized patients suffered from immunodeficiency affecting the humoral compartment, likely explaining the lower B cell count and IgRT as risk factors. As previously reported by Shield et al., in IEI, chronic lung disease represented a risk factor for hospitalization [13]. The overall mortality for COVID in our cohort was comparable to that observed in the Italian general population (3.5 vs 2.5%; *p* 0.7246), and the mean age of deceased people was much lower in our cohort (48 ± 12.52 vs 80 years). We also compared the age stratified mortality, and we found that the mortality in the 50–60 age range considerable exceeds the mortality from 50 to 60 age group of the Italian population (14.3 vs 0.6%; *p* < 0.0001) [24] (https://www.epicentro.iss.it/coronavirus/bollettino/Bollettino-sorveglianza-integrata-COVID-19_15-dicembre-2021.pdf). This might be due to the fact that compared to the general population IEI patients have more comorbidities, usually appearing at a younger age. Almost half of the patients in our cohort had preexisting lung disease. No-one under the age of 30 in the IEI cohort died. This is in keeping with other studies that suggest age is the biggest risk factor.

In the general population, the average time to recovery from COVID-19 is highly variable and depends on age and pre-existing comorbidities in addition to illness severity [7, 8]. Individuals with mild infection are expected to recover relatively quickly (e.g., within 2 weeks), whereas many individuals with severe disease have a longer time to recovery (e.g., 2 to 3 months) [7, 8, 11, 12, 25, 26]. In our cohort, we observed the persistence of symptoms beyond 3 weeks in 16.85% of the cases, and, as also observed in the general population, the occurrence of prolonged infection was significantly higher in hospitalized vs non-hospitalized patients (52.63 vs 5.5%, *p* < 0.0001). Similarly, a longer viral shedding was observed in hospitalized compared to non-hospitalized patients. Among the patients with prolonged infection, all but one patient with SIgAD were under IgRT, and patients with profound humoral immunodeficiency also showed a longer viral shedding compared to other IEI. This confirms what reported in previous studies from the literature showing that persistent infection or relapsing–remitting infection is associated with profound humoral immunodeficiency and the failure to make de novo humoral responses to SARS-CoV-2 [27]. In one patient with XLA from our cohort, readmission was required for the development of worsening dyspnea after a complete resolution of acute COVID-19 phase and negativization of the PRC on the nasopharyngeal swab. A few days after readmission, the nasopharyngeal swab became positive again, and the resolution of the symptoms was obtained with remdesivir and casirivimab/imdevimab [28]. No conclusive data are available on the management of patients with persisting COVID-19 symptoms. Treatment with corticosteroids has been shown to be beneficial in a subset of patients with post-COVID inflammatory lung disease [29]. Longer or multiple courses of remdesivir have been shown to be effective in persistent SARS-CoV-2 pneumonitis in humoral immune defects [30–32]. The effectiveness of passive antibody administration, including convalescent plasma and monoclonal antibodies, in COVID-19 is still debated, and information on the use of these drugs in immunodeficient patients is still limited. In a recent randomized trial, Simonovich et al. showed no difference in clinical outcome and mortality between convalescent plasma and placebo in the treatment of adult patients with severe SARS-CoV-2 pneumonia [33]. The European Medicines Agency recommend the use of monoclonal antibodies in patients with confirmed COVID-19 who do not require supplemental oxygen and are at high risk of progressing to severe COVID-19. In fact, in a large double-blind study, monoclonal antibody cocktail has been shown to be effective in reducing the viral load especially in patients whose immune response had not yet been initiated [34]. Mira et al. showed rapid recovery of a SARS-CoV-2-infected XLA patient after infusion of COVID-19 convalescent plasma [35]. In our study, information on the treatment was available for 7 out of 10 hospitalized patients with prolonged infection. Two patients were treated with convalescent plasma, 2 were treated with casirivimab/imdevimab, 1 was treated with anakinra, and the remaining 2 were treated with steroids. In 3 cases, these treatments were used in combination with antiviral drugs.

The rapid diffusion of the pandemic prompted studies on the development of new vaccination strategies. Patients with disorders of the immune system were vaccinated with priority. However, information on the efficacy of the vaccine in this population is still limited. We evaluated seroconversion after natural infection in 30 cases. Due to the difference in the techniques used for the analysis in the different labs, we were not able to compare the titer among the patients. Seroconversion was observed in most cases (86.6%). As recently shown in papers looking at the seroconversion after SARS-CoV-2 vaccination in IEI [36, 37], we also observed seroconversion in 5 out of 6 patients with CVID under IgRT and in 1 out of 2 patients with Good’s syndrome under IgRT. This would indicate that, in CVID, a residual functionality of B cell is still available.

In conclusion, in this study, we reported on the clinical course and outcome of SARS-CoV-2 infection in a large cohort of IEI patients, identified over a long period of observation. The identification of the patients through different phases of the pandemics has shown that, similarly to the general population, the clinical course in these patients is often very mild or asymptomatic. In fact, because of the redundancy of the immune system, only a few immunodeficiencies are associated with an increased risk of severe COVID. These include autoimmune polyendocrine syndrome type 1 and the immunodeficiencies involving the type I IFN pathway [38–44]. The severity and mortality of SARS-CoV-2 infection in this cohort seem to be related to the higher prevalence of comorbidities that usually appear early in life. We also confirmed that patients with humoral defects are able to produce antibodies after the natural infection. However, in some cases, patients with humoral immunodeficiencies may develop persistent infection or relapsing–remitting infection.

## Supplementary Information

Below is the link to the electronic supplementary material.

Supplementary file1 (DOCX 63 KB)

## Data Availability

Data are available upon request. For further information, please contact: pignata@unina.it.
