# Supplementary material for: The Impact of SARS-CoV-2 Infection in Patients with Inborn Errors of Immunity: the Experience of the Italian Primary Immunodeficiencies Network (IPINet)
Source: J Clin Immunol. 2022 Apr 20;42(5):935–46. doi: 10.1007/s10875-022-01264-y (PMC9020753; doi:10.1007/s10875-022-01264-y)
Supplement: Supplementary file 1 — Supplementary file1 (DOCX 63 KB) [file 10875_2022_1264_MOESM1_ESM.docx]

**Supplemental Table** Main clinical features of the patients included in the study

| Pt. | IEI | Sex | Age (years) | Comorbidities | Therapy for IEI | COVID Symptoms | Duration of the symptoms (days) | Hospitalized | Duration of viral shedding (days) | Seroconversion | Outcome |
| --- | --- | --- | --- | --- | --- | --- | --- | --- | --- | --- | --- |
| 1 | WAS | M | 1.4 | Autoinflammatory vasculitis | GT | Asymptomatic | 0 | - | 36 | Yes | Alive |
| 2 | WAS | M | 17.1 | Erythrocytosis, Corneal damage post-CMV infection | GT | Asymptomatic | 0 | - | 11 | Yes | Alive |
| 3 | cDGS | M | 1 | Minimal left to right interatrial shunt, Nephrocalcinosis, Hypoparathyroidism, Areas of parenchymal lung consolidation | Thymus transplantation, SCIg, Trimethoprim/Sulfamethoxazole, Fluconazole, Acyclovir | Fever | 30 | + | 7 | No | Alive |
| 4 | pDGS | F | 13.8 | Autoimmune hypothyroidism | - | Fever | 3 | - | 30 | NA | Alive |
| 5 | UAD | M | 8.8 | Recurrent infections, Previous encephalytis | Antibiotic prophylaxis | Asymptomatic | 0 | - | 10 | NA | Alive |
| 6 | Congenital neutropenia | M | 26.2 | Overweight, Hepatic steatosis, Interstitial lung disease | HSCT | Fever, Asthenia | 10 | - | 16 | NA | Alive |
| 7 | APDS1 | M | 24 | Atypical mycobacteriosis, Intestinal Diffuse Large B-Cell Lymphoma | IVIg | Asymptomatic | 0 | - | NA | NA | Alive |
| 8 | AT | M | 13 | - | SCIg | Asymptomatic | 0 | - | NA | NA | Alive |
| 9 | pDGS | F | 7 | - | - | Fever | NA | - | 9 | NA | Alive |
| 10 | pDGS | F | 1.5 | History of interventricular defect and supraventricular paroxysmal tachycardia | - | Fever | 10 | - | 30 | NA | Alive |
| 11 | pDGS | F | 23 | Keratoconus, Hypoacusia, Congenital heart disease, Allergy, Bronchiectasis | - | Running nose/Sore throat | 2 | - | NA | NA | Alive |
| 12 | CD4 lymphopenia | M | 3 | Recurrent bronchospasm, Congenital heart disease, Epilepsy, Developmental delay | - | Fever, Cough | 2 | - | 13 | NA | Alive |
| 13 | UAD | M | 9 | - | - | Asymptomatic | 0 | - | 10 | NA | Alive |
| 14 | NK reduction | M | 15 | Ulcerative colitis, Recurrent herpes and warts | - | Cough, Asthenia | 7 | - | 37 | Yes | Alive |
| 15 | pDGS | F | 17 | Autoimmune thyroiditis, Interventricular defect | - | Asymptomatic | 0 | - | 17 | NA | Alive |
| 16 | SCID (NBAS deficiency) |  | 2.75 | Autoimmune enteropathy, Chronic cholestatic hepatitis, Ichthyosiform dermatosis, Congenital hypothyroidism, Growth hormone deficiency, Developmental delay, Pulmonary hypertension, bronchiectasis, nodules and Interstitial lung disease | Trimethoprim/sulfamethoxazole, Acyclovir, IVIg, Dupilumab, Hydrocortisone | Dyspnea | 30 | + | NA | Yes | Alive |
| 17 | CVID | M | 39 | Chronic immune mediated thrombocytopenia, Marginal zone lymphoma, Splenectomy, Lymphadenopathy, Superficial vein thrombosis, Nodules | IVIg, Clarithromycin | Cough, Asthenia | 7 | + | 33 | NA | Alive |
| 18 | CVID | F | 34 | Recurrent urticaria, Lung nodules, Interstitial lung disease | SCIg | Fever, Dyspnea, Diarrhea, Asthenia, Sore throat, back pain, Headache, Anosmia, Ageusia | 11 | + | 33 | NA | Alive |
| 19 | CVID | F | 15.7 | - | IVIg | Asymptomatic | 0 | - | 11 | NA | Alive |
| 20 | CVID | M | 14.3 | Mild cognitive disability, Complex congenital heart disease (Tricuspid atresia, Hypoplastic right ventricle, Interventricular defect, Pulmonary artery stenosis), Congestive hepatopathy (post-Fontan operation) | IVIg | Fever, Cough | 8 | - | 17 | NA | Alive |
| 21 | XLA | M | 6.9 | - | SCIg | Fever, Diarrhea | 1 | - | 17 | NA | Alive |
| 22 | XLA | M | 5.5 | - | IVIg | Asymptomatic | 0 | - | 11 | NA | Alive |
| 23 | ARA | M | 19.7 | Chronic sinusitis | SCIg | Fever | 13 | + | NA | NA | Alive |
| 24 | pDGS | F | 9.25 | Congenital heart disease (Interrupted aortic arch, Interatrial defect, Interventricular defect) | Trimethoprim/Sulfamethoxazole | Fever, Asthenia | 1 | - | 26 | NA | Alive |
| 25 | UAD | M | 17.6 | - | - | Fever | 1 | - | NA | Yes | Alive |
| 26 | Kabuki syndrome | F | 12 | Cerebral lymphoproliferation | SCIg, Mycophenolate | Fever, Sore throat | 2 | - | 46 | NA | Alive |
| 27 | APDS | M | 15 | Crohn disease, Cytopenia, Bronchiectasis | SCIg, Trimethoprim/Sulfamethoxazole | NA | NA | - | NA | NA | Alive |
| 28 | XLA | M | 18 | - | SCIg | Fever, Headache | NA | - | 25 | NA | Alive |
| 29 | Myd88 deficiency | M | 8.5 | Multiple abdominal abscess, Right brachio-crural hemiparesis, Increased IgE levels | IVIg, Trimethoprim/Sulfamethoxazole | Asymptomatic | 0 | - | NA | Yes | Alive |
| 30 | IEI with immune dysregulation | M | 13 | Type 1 diabetes mellitus, Hepatosplenomegaly, Mild cognitive disability, microcephaly, Bone anomalies, Hypercholesterolemia, Myopia | - | Asymptomatic | 0 | - | NA | Yes | Alive |
| 31 | HyperIgM | F | 24.7 | Bronchiectasis, Recurrent urinary tract infection, History of non-Hodgkin lymphoma | HSCT | Asymptomatic | 0 | - | NA | Yes | Alive |
| 32 | pDGS | M | 18.4 | Autoimmune thyroiditis, Mild thrombocytopenia, Hyperbilirubinemia, Vitamin D deficiency | - | Running nose | NA | - | NA | Yes | Alive |
| 33 | pDGS | F | 11.5 | Obesity, Cognitive disability | - | Cough, Headache, Dyspnea, Asthenia, Nausea, Arthralgia | 19 | - | NA | Yes | Alive |
| 34 | AT | M | 19.5 | Restrictive lung disease, Atelectasis | - | Fever, Cough, Dyspnea, Myalgia | 6 | - | NA | Yes | Alive |
| 35 | SCID | F | 15 | Cognitive disability, Cerebral atrophy, Hypoacusia, Scoliosis, Arthritis | HSCT | Asymptomatic | 0 | - | NA | Yes | Alive |
| 36 | UAD | F | 8.5 | Recurrent respiratory infections, Hypercholesterolemia | - | NA | NA | - | NA | Yes | Alive |
| 37 | pDGS | M | 1.6 | Hypoparathyroidism, Hypocalcemia, Congenital hypothyroidism, Bicuspid aorta | - | Asymptomatic | 0 | - | NA | Yes | Alive |
| 38 | EO-IBD | M | 2.25 | Dilated left ventricle | Methotrexate, Ustekinumab | NA | NA | - | NA | Yes | Alive |
| 39 | Neutropenia | F | 1.5 | Pulmonary valve stenosis, Interatrial comunication with left to right shunt, Corpus callosum hypoplasia, Left kidney with double district | - | Fever | 1 | - | 17 | Yes | Alive |
| 40 | CID | M | 16.3 | Bronchiectasis, Lung nodule, Intellectual disability | - | Asymptomatic | 0 | - | NA | Yes | Alive |
| 41 | CID | M | 19.5 | Ulcerative colitis | Mesalazine | Asymptomatic | 0 | - | NA | Yes | Alive |
| 42 | pDGS | M | 10.6 | Left aortic arch, Developmental delay | - | NA | NA | - | NA | NA | Alive |
| 43 | pDGS | F | 15.2 | Hyperthyroidism, Intellectual disability, Congenital heart disease | - | NA | NA | - | NA | Yes | Alive |
| 44 | SIgAD | M | 22.3 | History of chronic immune thrombocytopenia, Pyelectasis | - | Ageusia, Astenia | NA | - | 10 | NA | Alive |
| 45 | WAS | M | 10 | Scoliosis, Kaposi-like hemangioendothelioma | - | Asymptomatic | NA | - | NA | NA | Alive |
| 46 | CVID | M | 42 | - | SCIg | Fever, Cough, Dyspnea | 60 | + | 79 | NA | Alive |
| 47 | Good syndrome | M | 51 | Obesity, Thymoma, Hypertension, Bronchiectasis | SCIg, Chemo- and radiotherapy for thymoma | Fever, Cough, Dyspnea | NA | + | NA | NA | Dead |
| 48 | CVID | F | 40 | Obesity, Interstitial lung disease | SCIg, Inhalatory corticosteroid, Montelukast | Fever | 90 | - | 76 | NA | Alive |
| 49 | CVID | M | 45 | GLILD, Autoimmune enteropathy, Bronchiectasis, Nodules | SCIg | Fever, Cough | 14 | - | NA | NA | Alive |
| 50 | CVID | M | 13 | - | IVIG | Fever | 5 | - | 18 | NA | Alive |
| 51 | SIgAD | M | 19 | - | - | Asymptomatic | 0 | - | 27 | NA | Alive |
| 52 | Idiopathic CD4 lymphopenia | F | 32 | - | - | NA | NA | - | NA | NA | Alive |
| 53 | SIgAD | M | 19 | - | - | Fever, Asthenia | 7 | - | NA | NA | Alive |
| 54 | SIgAD | M | 19 | Celiac disease | - | NA | NA | - | NA | NA | Alive |
| 55 | CVID | F | 30 | Neonatal encephalopathy, Disability, Polycystic ovary syndrome, Bronchiectasis | SCIg, Inhalatory corticosteroid | NA | NA | + | NA | NA | Dead |
| 56 | CVID | F | 63 | Autoimmune hepatitis, Nodules | SCIg, Azathioprine | Fever, Diarrhea | 14 | - | 28 | Yes | Alive |
| 57 | CVID | F | 66 | - | IVIg | Asthenia | 7 | - | 22 | NA | Alive |
| 58 | CVID | F | 52 | Autoimmune thyroiditis | IVIg | Fever, Cough, Diarrhea, Asthenia | 6 | - | 22 | NA | Alive |
| 59 | Good Syndrome | M | 53 | Thymoma, Myasthenia | Antibiotic prophylaxis | Fever, Cough, Diarrhea, Asthenia | 10 | - | 14 | NA | Alive |
| 60 | CVID | M | 52 | Bilateral lung transplant, Chronic respiratory failure, Chronic transplant rejection, Previous GLILD, Interstitial lung disease and bronchiectasis | SCIg, O2 therapy, Noninvasive mechanical ventilation, Prednisone, Everolimus, Cyclosporine, Trimethoprim/Sulfamethoxazole, Isavuconazole, Inhalatory corticosteroids and Tobramicine | NA | NA | + | NA | NA | Dead |
| 61 | CVID | M | 45 | Diabetes mellitus, Hypertension, Obesity, Atrophic gastritis | SCIg | NA | NA | - | NA | NA | Alive |
| 62 | XLA | M | 32 | Bronchiectasis | SCIg, Azitromicine | Fever, Cough, Dyspnea | 48 | + | 45 | NA | Alive |
| 63 | XLA | M | 19 | - | IVIg | Asymptomatic | 5 | - | 36 | NA | Alive |
| 64 | CVID | M | 57 | Eosinophilic granulomatosis with polyangiitis, Hypertension, Impaired glucose tolerance, Atelectasis | IVIg, Prednisone, Inhalatory steroid | Fever, Cough, Dyspnea, Asthenia | 34 | + | 34 | NA | Alive |
| 65 | CVID | M | 59 | Chronic obstructive pulmonary disease, Transuretral resection of prostate for benign prostate hypertrophy, Sinusitis, Hepatic steatosis, Bronchiectasis | SCIg, Inhalatory steroids | Cough, Dyspnea | 7 | - | 16 | NA | Alive |
| 66 | CVID | F | 49 | History of melanoma and breast cancer treated with radiotherapy, Bronchiectasis | SCIg | Fever | 7 | - | 21 | NA | Alive |
| 67 | CVID | F | 46 | Bronchiectasis, Interstitial lung disease, Chronic immune mediated thrombocytopenia | IVIg | NA | NA | - | NA | Yes | Alive |
| 68 | CVID | F | 27 | - | SCIg | Asymptomatic | 0 | - | 15 | Yes | Alive |
| 69 | Good Syndrome | M | 63 | Hypertension, Lung nodules | IVIg, Azitromicine, Prednisone | Fever, Cough, Dyspnea | 60 | + | 59 | NA | Alive |
| 70 | CVID | M | 54 | Chronic immune mediated thrombocytopenia, Interstitial lung disease, nodules | SCIg | Fever, Cough, Dyspnea | 11 | + | 10 | NA | Alive |
| 71 | CVID | F | 58 | Bronchiectasis, Chronic renal failure, Hypertension | SCIg, Inhalatory steroids | NA | NA | - | NA | NA | Alive |
| 72 | CVID | F | 55 | Bronchiectasis, Interstitial lung disease | IVIg | Asymptomatic | 0 | - | 9 | NA | Alive |
| 73 | CVID | M | 49 | Bronchiectasis, interstitial lung disease, Chronic immune mediated thrombocytopenia | IVIg | Asymptomatic | 0 | - | 9 | NA | Alive |
| 74 | CVID | M | 50 | Liver failure, Nodules | IVIg, Metronidazole | Asymptomatic | 0 | - | NA | NA | Alive |
| 75 | CVID | M | 48 | Bronchiectasis | IVIg, Azitromicine | Fever, Cough, Dyspnea | 5 | - | 23 | NA | Alive |
| 76 | CVID | F | 69 | Bronchiectasis, interstitial lung disease, nodules | IVIg, Mycophenolate mofetil, Azitromicine, | Fever, Asthenia | 5 | - | 20 | NA | Alive |
| 77 | UAD | M | 80 | Hypertension, Chronic obstructive pulmonary disease, Chronic renal failure, Heart failure | IVIg | Fever, Cough, Dyspnea | 40 | + | 51 | NA | Alive |
| 78 | UAD | F | 71 | Hypertension, Obesity, Interstitial lung disease | IVIg, Inhalatory cotricosteroids | Fever, Dyspnea, Asthenia, Anosmia | 25 | - | 27 | NA | Alive |
| 79 | CVID | M | 27 | Bronchiectasis | - | NA | NA | - | NA | NA | Alive |
| 80 | CVID | F | 44 | Interstitial lung disease | SCIg | Asymptomatic | 0 | - | 34 | NA | Alive |
| 81 | UAD | M | 65 | Prostate cancer, Epilepsy, Hypertension, Chronic obstructive pulmonary disease, Obesity | IVIg | Asymptomatic | 0 | - | 34 | NA | Alive |
| 82 | CVID | F | 47 | Diabetes, Interstitial lung disease | SCIg | NA | NA | + | NA | NA | Alive |
| 83 | CVID | M | 33 | Autoimmune hemolytic anemia, Interstitial lung disease | SCIg | Asymptomatic | 0 | - | NA | NA | Alive |
| 84 | CVID | F | 64 | Bronchiectasis, hypertension, Chronic immune mediated thrombocytopenia, GLILD | IVIg, Prednisone | Arthralgia, Anosmia | 3 | - | 12 | NA | Alive |
| 85 | CVID | F | 45 | Bronchiectasis, Hypertension | SCIg | Fever | 30 | - | 43 | Yes | Alive |
| 86 | CVID | F | 41 | Atelctasis | SCIg | NA | NA | - | NA | NA | Alive |
| 87 | CVID | M | 53 | Colon cancer, Nodules | SCIg | Asymptomatic | 0 | - | 16 | Yes | Alive |
| 88 | CVID | M | 55 | Hypertension, Chronic obstructive pulmonary disease, Interstitial lung disease, nodules | IVIg | Asymptomatic | 0 | + | 51 | NA | Alive |
| 89 | CVID | M | 46 | Chronic sinusitis, Interstitial lung disease | IVIg | Asymptomatic | 0 | - | 30 | NA | Alive |
| 90 | ARA | M | 53 | Chronic obstructive pulmonary disease, Nodules, Hypoacusia | SCIg | Asymptomatic | 0 | + | 6 | No | Alive |
| 91 | CVID | F | 59 | Chronic sinusitis | SCIg, Inhalatory corticosteroid | Fever, Cough, Dyspnea | 20 | + | NA | NA | Dead |
| 92 | SIgAD | F | 33 | Allergy | - | Fever, Dyspnea | 15 | + | 45 | Yes | Alive |
| 93 | CVID | M | 38 | Chronic immune mediated thrombocytopenia, Autoimmune hemolitic anemia, Bronchietasis, History of stroke | IVIg, Prednisone, Azitromicine | Fever, Cough, Dyspnea, Asthenia | 30 | - | 23 | NA | Alive |
| 94 | CVID | F | 49 | Splenectomy, GLILD, Bronchiectasis, Nodules, Chronic lymphocytosis | IVIg | Fever, Cough, Dyspnea | 40 | + | 40 | No | Alive |
| 95 | CVID | M | 30 | Ulcerative colitis | SCIg, Mesalazine | Fever | 6 | - | 23 | NA | Alive |
| 96 | CVID | M | 55 | Ischemic cardiopathy, Hypertension, Thyroid disease | IVIg | Asymptomatic | 0 | - | 81 | NA | Alive |
| 97 | CVID | F | 47 | Chronic obstructive pulmonary disease, Bronctiectasis | SCIg, Inhalatory corticosteroids | Fever, Cough, Dyspnea | 60 | + | 75 | NA | Alive |
| 98 | CVID | M | 57 | Chronic immune mediated thrombocytopenia, Nodules | SCIg | Fever | 18 | - | 77 | NA | Alive |
| 99 | CVID | M | 33 | History of Non-Hodgkin lymphoma, Bronchiectasis, Interstitial lung disease, Nodules | SCIg | Fever, Cough | 7 | + | NA | NA | Alive |
| 100 | CVID | M | 52 | Chronic sinusitis, Lymphoid nodular hyperplasia, Vitiligo, History of bacterial meningoencephalitis and viral encephalitis, Bronchiectasis, Interstitial lung disease, Nodules | SCIg, Azitromicine | Asymptomatic | 0 | - | NA | NA | Alive |
| 101 | CVID | M | 60 | Chronic gastritis with intestinal metaplasia, Interstitial lung disease | SCIg | Fever, Asthenia | NA | - | NA | NA | Alive |
| 102 | XLA | M | 54 | Bronchiectasis, Atelectasis, Nodules, Chronic prostatitis, Chronic sinusitis | IVIg | Fever, Cough, Dyspnea, Asthenia | NA | + | NA | NA | Alive |
| 103 | CVID | F | 56 | Nodules | IVIg | Fever, Diarrhea | 3 | - | NA | NA | Alive |
| 104 | CVID | F | 55 | Lymphoid nodular hyperplasia | SCIg | Asymptomatic | 0 | - | NA | NA | Alive |
| 105 | CVID | M | 46 | Lymphoid nodular hyperplasia, Splenomegaly, Bronchiectasis | SCIg | Fever, Cough, Asthenia | 7 | - | NA | NA | Alive |
| 106 | CVID | M | 54 | Lymphoid nodular hyperplasia | IVIg | Fever, Asthenia | 7 | - | NA | NA | Alive |
| 107 | CVID | F | 45 | Celiac disease, Nodules | fSCIg | Asymptomatic | 3 | - | NA | NA | Alive |
| 108 | SIgAD | M | 29 | Overweight | - | Cough, Dyspnea, Diarrhea, Asthenia | 15 | - | NA | NA | Alive |
| 109 | SIgAD | F | 64 | Food allergy | - | Fever, Cough, Dyspnea, Diarrhea, Asthenia | 30 | - | NA | NA | Alive |
| 110 | SIgAD | F | 32 | Allergy | - | Cough | 2 | - | NA | NA | Alive |
| 111 | CVID | F | 39 | GLILD, Bronchiectasis, Nodules, Splenomegaly, Lymphadenopathy immune trombocytopenia, Psoriasis | SCIg | Diarrhea, Asthenia | NA | - | NA | NA | Alive |
| 112 | CVID | F | 32 | - | SCIg | Fever, Asthenia | 15 | - | NA | NA | Alive |
| 113 | XLA | M | 28 | Bronchiectasis, Interstitial lung disease | IVIg | Fever, Cough, Dyspnea, Diarrhea, Asthenia | 52 | + | NA | NA | Alive |
| 114 | CVID | F | 29 | Bronchiectasis | SCIg | Fever, Cough | NA | - | 36 | NA | Alive |

IEI, Inborn error of immunity; WAS, Wiskott-Aldrich Syndrome; GT, Gene therapy; cDGS, complete Di George syndrome; SCIg, Subcutaneous immunoglobulin; pDGS, partial Di George syndrome; UAD, Unclassified antibody deficiency; HSCT, Hematopoietic stem cell transplantation; APDS1, Activated PI3K delta syndrome - type 1; IVIg, Intravenous immunoglobulin; NA, Not available; AT, Ataxia Telangiectasia; NK, Natural Killer; SCID, Severe combined immunodeficiency; NBAS, Neuroblastoma amplified sequence; CVID, Common variable immunodeficiency; XLA, X-linked agammaglobulinemia; ARA, Autosomal recessive agammaglobulinemia; SIgAD, Selective IgA deficiency; EO-IBD, Early onset inflammatory bowel disease; CID, Combined immunodeficiency; GLILD, Granulomatous interstitial lung disease.
